# Vitiligo auto‐immune response upon oxidative stress‐related mitochondrial DNA release opens up new therapeutic strategies

**DOI:** 10.1002/ctm2.1810

**Published:** 2024-08-07

**Authors:** Ana C. B. Sant'Anna‐Silva, Thomas Botton, Andrea Rossi, Jochen Dobner, Hanene Bzioueche, Nguyen Thach, Lauriane Blot, Sophie Pagnotta, Konrad Kleszczynski, Kerstin Steinbrink, Nathalie M. Mazure, Stéphane Rocchi, Jean Krutmann, Thierry Passeron, Meri K. Tulic

**Affiliations:** ^1^ Université Côte d'Azur, INSERM U1065, C3M Nice France; ^2^ IUF‐Leibniz Research Institute for Environmental Medicine Düsseldorf Germany; ^3^ Common Centre of Applied Microscopy (CCMA) Université Côte d'Azur Nice France; ^4^ Department of Dermatology University Hospital Münster Münster Germany; ^5^ Medical Faculty Heinrich‐Heine‐University Düsseldorf Germany; ^6^ Department of Dermatology Université Côte d'Azur, CHU Nice Nice France

Dear Editor

Vitiligo is a multifactorial auto‐immune disease that causes melanocyte loss, leading to skin and hair depigmentation. Despite the efficacy of JAK inhibitors in blunting the anti‐melanocyte immune response, skin re‐pigmentation still takes more than 1–2 years, and most patients do not attain a complete response, thus additional therapeutic options are needed.

We have previously shown innate immunity to play a critical role in melanocyte destruction^1^ and have identified the presence of mitochondrial DNA (mtDNA) in the skin of some vitiligo patients that strongly correlated with enrichment of opportunistic or pathogenic bacteria, reduced presence of commensal strains and elevated blood levels of pro‐inflammatory cytokines.^2^ Therefore, we wondered whether instead of being a by‐product of melanocyte destruction, mtDNA may be involved in vitiligo pathogenesis.

mtDNA amplicon sequencing of primary melanocytes from 14 healthy donors and 16 patients with non‐active vitiligo identified five vitiligo samples with supraphysiological number of mtDNA variants (termed high variant load; Vitiligo HV) compared to vitiligo melanocytes with low variant load (Vitiligo LV; Figure 1A). The number of mtDNA variants was not correlated with subject's age suggesting that they were not randomly accumulating over time (Supporting Information Figure 1A–D) but rather driven by elevated mitochondrial hydroxyl radical levels as supported by frequent C > T and T > C transitions (Supporting Information Figure 1E). The number of mtDNA variants positively correlated with the release of double‐stranded (ds)DNA in melanocyte culture supernatant (Supporting Information Figure 1F). Thus, higher amounts of dsDNA were found in the supernatant of Vitiligo HV melanocytes with decreased mtDNA in their mitochondria (Figure 1B,C). Likewise, increased amounts of transcripts from the mitochondrial encoded genes *MT‐ND1* and *MT‐CO1* were found in Vitiligo HV culture supernatants (Figure 1D), positively correlating with the number of mtDNA variants (Figure 1E). Noticeably, mtDNA variants observed in melanocytes were of somatic origin as they were less common in matching peripheral blood mononuclear cell (PBMCs) (Figure 1F).

**FIGURE 1:**
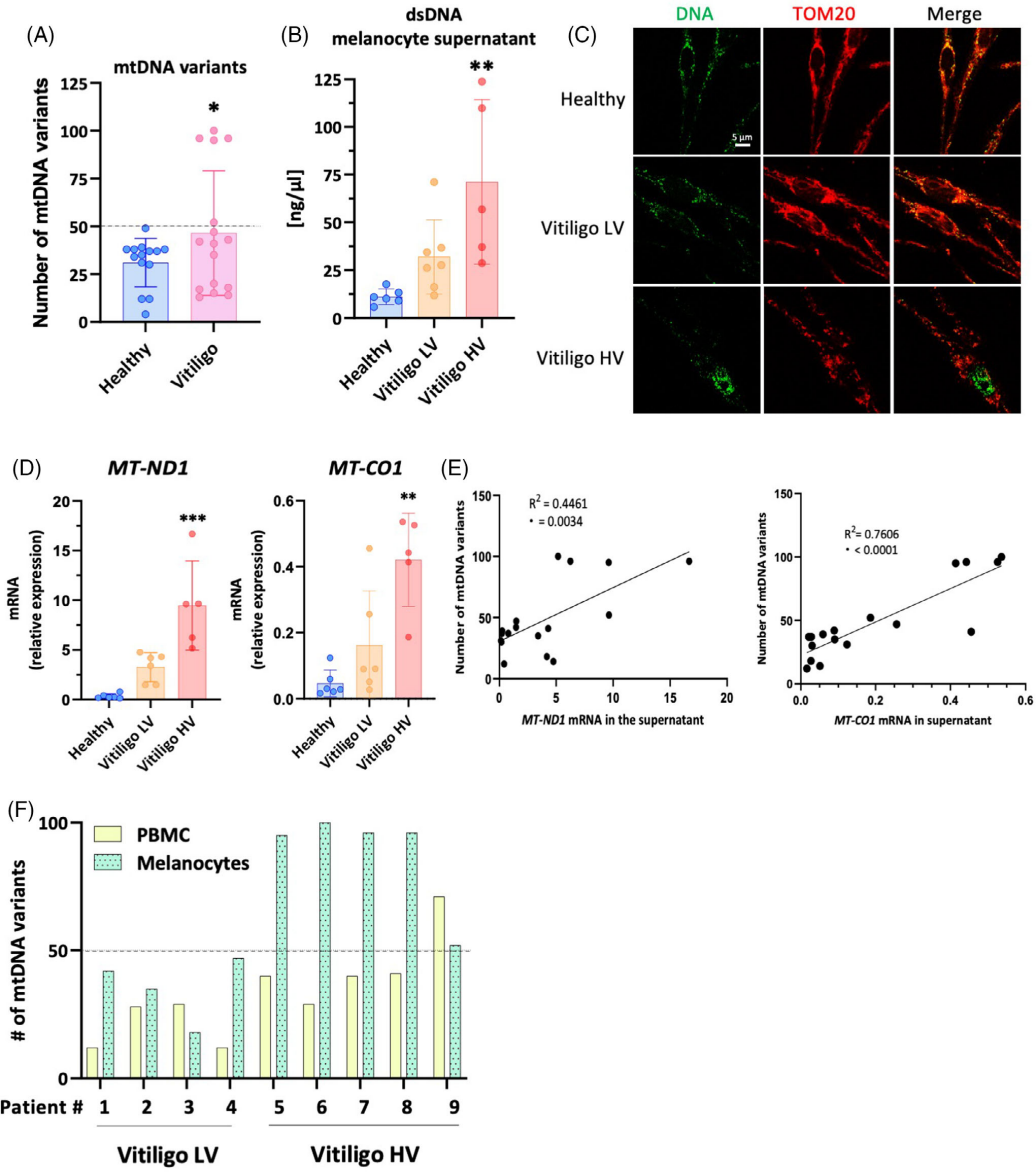
Some vitiligo melanocytes present elevated frequency of mitochondrial DNA (mtDNA) variants associated with mitochondrial nucleic acids DNA release. (A) Number of mtDNA variants in melanocyte cultures from healthy donors (*n* = 14) and non‐active vitiligo patients (*n* = 16); (B) Measurement of double‐stranded DNA (dsDNA) in the culture supernatant of melanocytes from healthy (*n* = 6), Vitiligo low variants (LVs; *n* = 7) and Vitiligo high variants (HV; *n* = 5); (C) Representative images of melanocytes from healthy donors, Vitiligo LV or Vitiligo HV were immunostained for DNA (green) and the outer membrane mitochondrial marker TOM20 (red; *n* = 3 for each condition). (D) Relative abundance of *MT‐ND1* or *MT‐CO1* transcript in melanocyte culture supernatant (*n* = 6 healthy, 6 LV and 5 HV measured in at least three technical triplicates). (E) Correlation between the number of mtDNA variants present melanocytes and the amount of *MT‐ND1* or *MT‐CO1* transcripts detected in their culture supernatant. (F) Number of mtDNA variants found in melanocytes or PBMCs of four LV and five HV vitiligo melanocytes cultures. All data represent mean  ±  SD. One‐way analysis of variance (ANOVA) compared to healthy controls. *p < .05; **p < .01; ***p < .001.

To investigate whether mtDNA release could originate from mitochondrial impairment, we first demonstrated decreased mitochondrial complexes protein subunits expression in both Vitiligo LV and HV compared to healthy controls (Supporting Information Figure 2A,B). However, only Vitiligo HV showed mitochondrial network with greater branching and mass; the latter positively correlating with the number of mtDNA variants (Figure 2A–C). Furthermore, Vitiligo HV compensated their oxidative damage by increasing their total adenosine triphosphate (ATP) production, oxygen consumption rate (OCR) and ATP‐coupled respiration compared to Vitiligo LV and healthy melanocytes (Figure 2D–F). Consistently, Vitiligo HV have higher dependency on mitochondrial function rather than glycolysis for their ATP production (Figure 2G), resulting in elevated production of mitochondrial reactive oxygen species (ROS; Figure 2H), later which was further exacerbated by the pro‐oxidant menadione (Figure 2I). We found two rare single nucleotide polymorphism (SNPs) in catalase (rs371396899 and rs17268652) to be tightly associated with the Vitiligo HV phenotype (Supporting Information Figure 2C). Functional assay confirmed lower catalase activity in Vitiligo HV melanocytes (Figure 2J), supporting lower ROS scavenging capacity leading to greater oxidative stress. Our observations are in line with previous reports demonstrating that mitochondrial oxidative stress can disrupt mtDNA integrity and facilitate its release into the cytoplasm and the extracellular space.^3^

**FIGURE 2:**
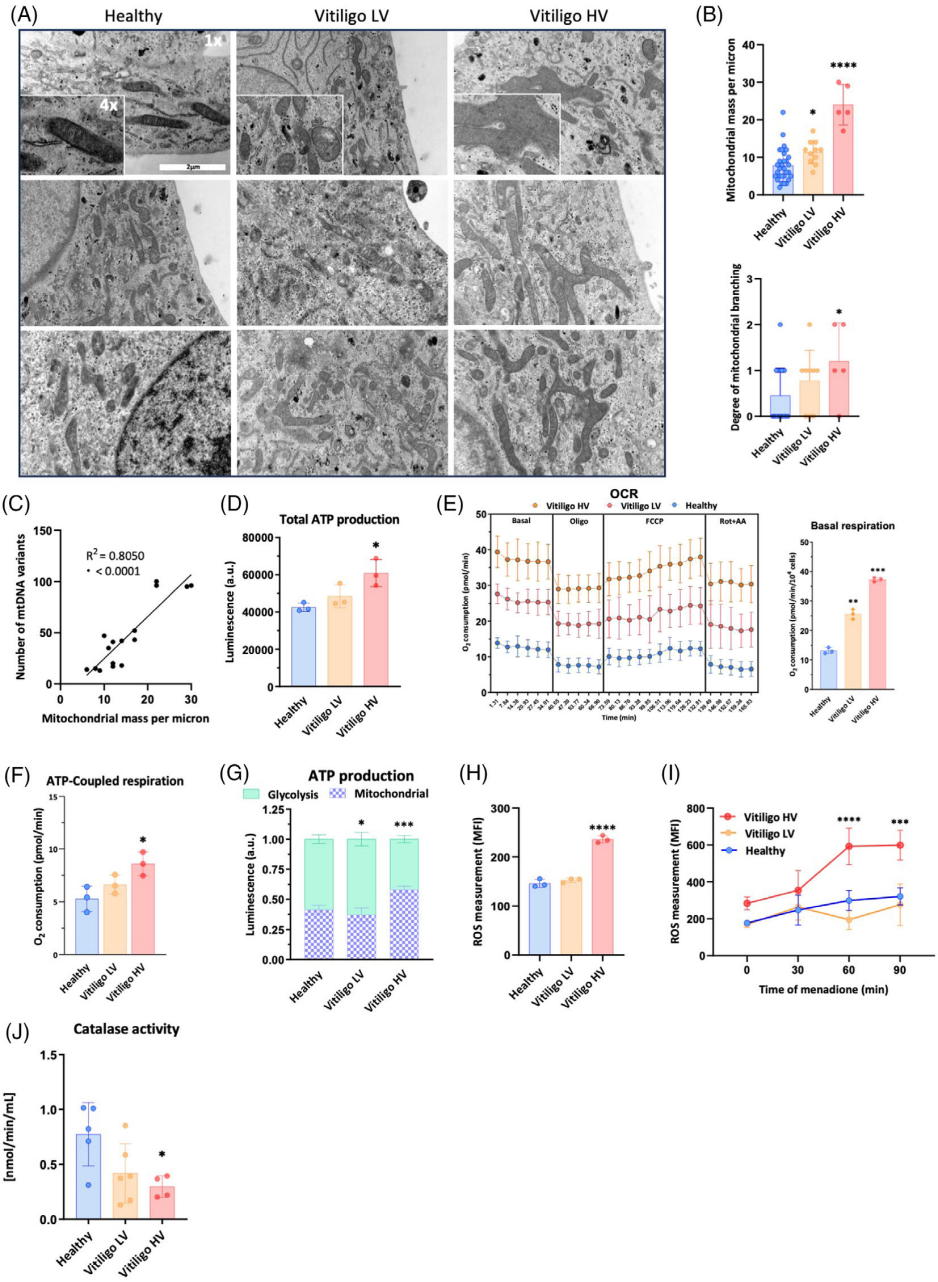
Vitiligo high variant (HV) melanocytes display increased mitochondrial function, elevated reactive oxygen species (ROS) production and are associated with decreased peripheral catalase activity. (A) Representative transmission electron microscopy (TEM) images from three independent cultures of healthy, Vitiligo low variant (LV) and Vitiligo high variant (HV) melanocytes. Scale bar represents 2 µm (1×) and zoomed image is 4× the original picture; (B) Quantification of mitochondrial mass (*n* = 30 healthy, 11 LV and 5 HV melanocyte cultures) and scoring of mitochondrial branching (*n* = 20 healthy, 9 LV and 5 HV) per cell; (C) Correlation between mitochondrial mass and the number of mitochondrial DNA (mtDNA) variants in melanocytes; (D) Total ATP production; (E) Left panel: oxygen consumption rate. Cells were treated with oligomycin (1 µM), carbonyl cyanide p‐trifluoro methoxy phenylhydrazone (FCCP; 20 nM titrations), rotenone (1 µM) and antimycin A (1 µM). Right panel: quantification of basal respiration. (F) ATP‐coupled respiration; (G) Measurement of ATP contribution from glycolysis and mitochondria; (H) ROS measurement at baseline and (I) in response to oxidative stress induced by 50 µM menadione; (J) Catalase activity assay in matched PBMCs (*n* = 5 healthy, 6 LV and 4 HV). Experiments from panels (D) to (I) were performed on three independent melanocyte cultures for each condition in at least three technical triplicates. All data represent mean  ±  SD. One‐way analysis of variance (ANOVA) compared to healthy controls. *p < .05; **p < .01; ***p < .001; ****p < .0001.

mtDNA release in the cytosol has been linked to activation of the cyclic GMP‐AMP synthase‐stimulator of interferon genes (cGAS‐STING) pathway which activates immune responses.^3^ Consistent with these observations, we demonstrated an induction of phosphorylated TANK‐binding kinase 1 (p‐TBK1) downstream of the cGAS‐STING pathway in Vitiligo HV but not in Vitiligo LV or healthy melanocytes, along with an induction of interferon regulatory factor (IRF)3, IRF7 and the NF‐κB pathway (Figure 3A,B). Downstream of these transcription factors critical for chemokines and cytokines production, Vitiligo HV demonstrated elevated levels of interleukin (IL)1‐β, IL‐18, type I interferons (IFNα/β), CXCL9 and CXCL10 in the culture supernatants resulting in strong PBMC recruitment when the same HV secretome was used as a chemoattractant (Figure 3C,D).

**FIGURE 3:**
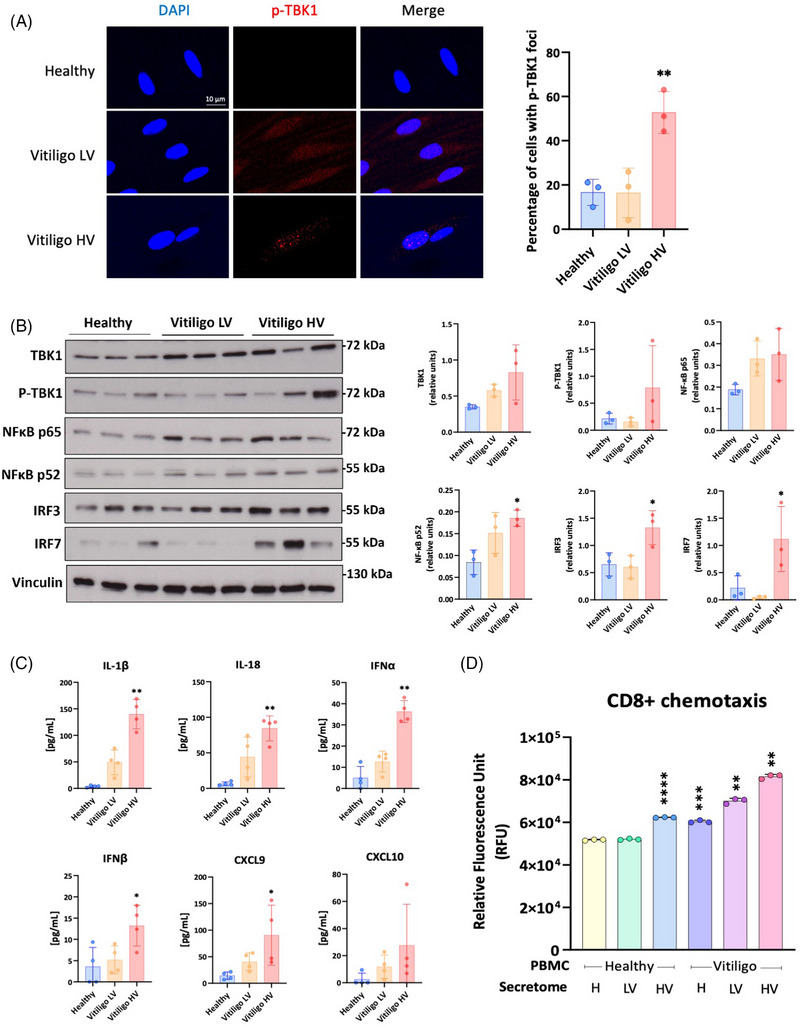
Vitiligo high variant (HV) triggers inflammatory response via the cyclic GMP‐AMP synthase‐stimulator of interferon genes (cGAS‐STING) pathway. (A) Melanocytes from healthy donors, Vitiligo low variant (LV) or high variant (HV) were immunostained for phosphorylated TANK‐binding kinase 1 (p‐TBK1) and the percentage of cells with p‐TBK1 foci was quantified (*n* = 3 melanocyte cultures per condition and at least 45 cells per datapoint). (B) Immunoblot analysis of melanocyte lysates for STING, TBK1, p‐TBK1, NF‐kB p65, NF‐kB p100/p52, interferon regulatory factor (IRF)3 and IRF7 (*n* = 3 melanocyte cultures per group). Vinculin was used as a loading control. (C) ELISA assay from supernatants measuring interleukin (IL)‐1β, IL‐18, interferon (IFN)α, IFNβ, CXCL9 and CXCL10 (*n* = 3 melanocyte cultures per condition measured in technical triplicates). (D) Chemotaxis assay of CD8+ cells from healthy donors or vitiligo patients using culture supernatants from healthy melanocytes, Vitiligo LV or HV melanocytes (*n* = 3 melanocyte cultures per condition measured in technical triplicates). All data represent mean  ±  SD. One‐way analysis of variance (ANOVA) compared to healthy controls. *p < .05; **p < .01; ***p < .001; ****p < .0001.

We further demonstrated the therapeutic potential of targeting this new signalling cascade in vitiligo by showing that mitochondrial‐specific ROS scavenger recombinant superoxide dismutase 2 (SOD2) and nuclear factor erythroid 2‐related factor 2 (NRF2) activators (dimethyl fumarate, DMF or NK‐252) decrease dsDNA release, cytokine and chemokine production specifically in Vitiligo HV (Figure 4A–H). Conversely, non‐specific antioxidant vitamin C or cytoplasmic antioxidant SOD1 had a modest effect on dsDNA release and failed to reduce cytokine and chemokine production (Supporting Information Figure 3A,B). Next, we tried to prevent mtDNA cytoplasm release by using the VDAC‐1 oligomerisation inhibitor VBIT‐4 which proved ineffective (Figure 4A–H). Finally, the TBK1 inhibitor GSK8612 did not affect dsDNA release but inhibited the melanocyte secretome (except for IL‐1β and CCL19) that is downstream of NF‐κB and directly activated by STING. Nevertheless, the secretome of Vitiligo HV melanocytes treated with GSK8612, like recombinant SOD2, inhibited the CD8+ T‐cell recruitment (Figure 4I,J). In future studies, it would be of interest to see whether modulating those proteins targeted by NRF2 activators (including natural products such as CYP11A1‐derived vitamin D3 hydroxyderivatives or melatonin^4^) can restore mitochondrial function in melanocytes.

**FIGURE 4:**
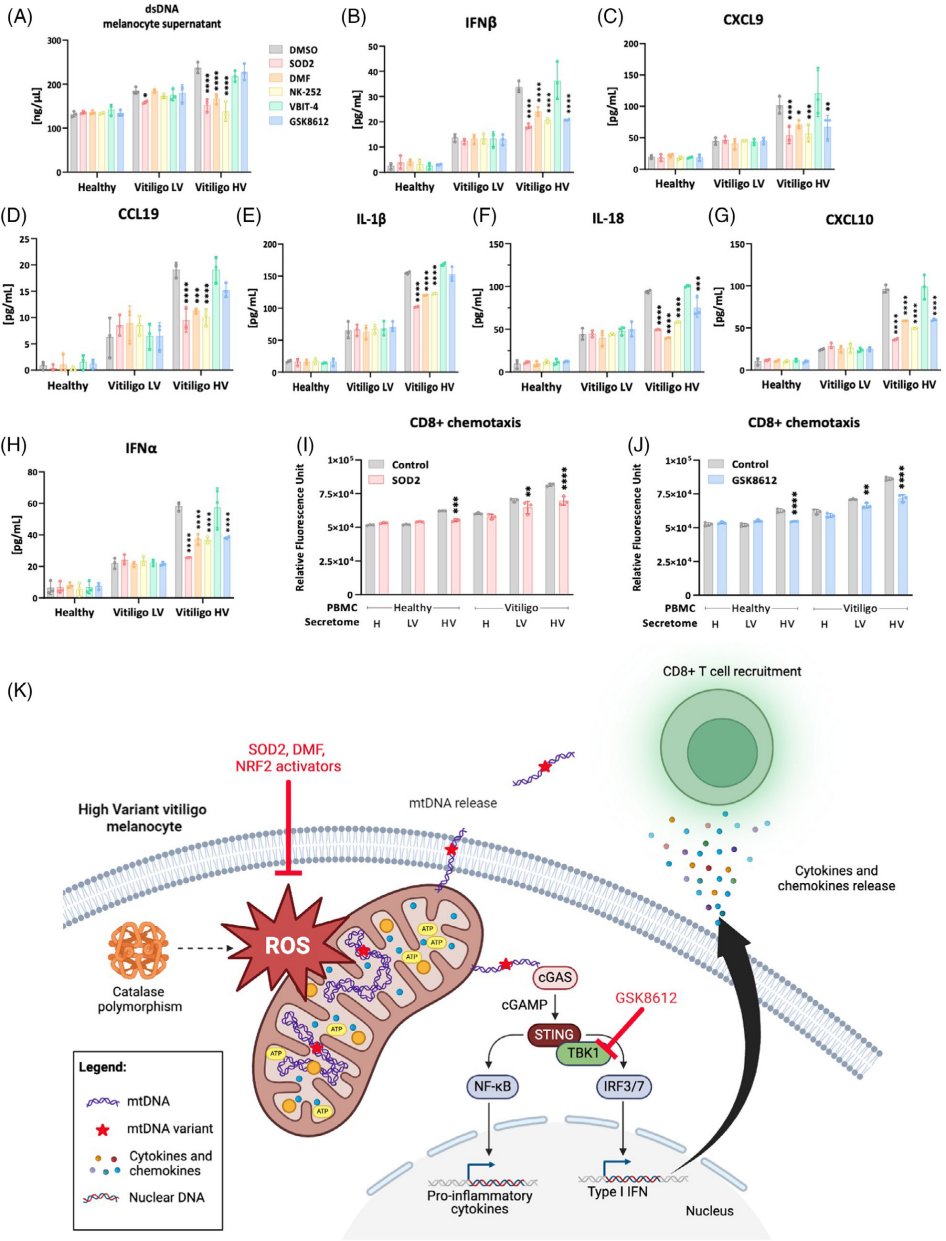
Preventing mitochondrial DNA (mtDNA) release or sensing blunts inflammatory response responsible from immune cells recruitment. (A) Measurement of double‐stranded DNA (dsDNA) and a panel of cytokines (B–E) and chemokines (F–H) in the supernatant of melanocytes treated with nuclear factor erythroid 2‐related factor 2 (NRF2) activators dimethyl fumarate (DMF; 50 µM) or NK‐252 (100 µM), recombinant mitochondrial antioxidant superoxide dismutase 2 (SOD2; 50 µg/mL), VDAC‐1 inhibitor VBIT‐4 (20 µM) or TANK‐binding kinase 1 (TBK1) inhibitor GSK8612 (10 µM); (I, J) Chemotaxis assay of CD8+ cells from healthy or vitiligo subjects using culture supernatants form healthy melanocytes, Vitiligo low variant (LV) or high variant (HV) melanocytes previously treated (or not) with SOD2 (50 µg/mL) (I) or TBK1 inhibitor GSK8612 (10 µM) (J). All data represent mean  ±  SD. *n* = 3 melanocyte cultures per condition measured in at least three technical triplicates. Two‐way analysis of variance (ANOVA) compared to healthy controls. *p < .05; **p < .01; ***p < .001; ****p < .0001. (K) Schematic summary of our findings illustrating the induction of vitiligo auto‐immune response upon oxidative stress‐related mitochondrial DNA release. A subset of vitiligo subjects harbouring polymorphisms in their CAT gene have decreased catalase activity and elevated level of reactive oxygen species (ROS), which was associated with supraphysiological levels of mtDNA variants in their melanocytes. In this context, release of mtDNA triggers the activation of TBK1 downstream of the cyclic GMP‐AMP synthase‐stimulator of interferon genes (cGAS‐STING) pathway and the induction of pro‐inflammatory cytokines and chemokines responsible for the attraction of CD8+ cells and the initiation of an auto‐immune response against melanocytes. This process can be blocked at various levels using mitochondrial antioxidant SOD2, NRF2 activators or TBK1 inhibitors. Created with BioRender.com.

Somatic mtDNA variants have been reported in aging and neurodegenerative disorders^5^ but there are very limited data in auto‐immune diseases. Somatic mtDNA variants in synoviocytes have been found to be increased in rheumatoid arthritis.^6^ Interestingly, MELAS (mitochondrial encephalopathy, lactic acidosis and stroke‐like episodes) syndrome is principally due to a mutation in mtDNA and it is associated with a 10‐fold increased risk of developing vitiligo compared to the general population.^7^ Similarly, in Vogt–Koyanagi–Harada syndrome where mitochondrial oxidative stress plays a key role^8^ and in Kabuki syndrome^9^ where loss of function of the histone methyltransferase KMT2D leads to a dysregulation of mitochondrial respiration; frequency of vitiligo is increased compared to the general population. Furthermore, defective mitophagy in melanocytes seems to be linked to activation of autoimmunity in vitiligo.^10^ Together with our novel results, there is now strong evidence placing the dysregulation of mitochondrial homeostasis as a cornerstone in vitiligo initiation.

In conclusion, we have demonstrated a cascade of events linking the mitochondrial oxidative stress occurring in vitiligo melanocytes to accumulation of mtDNA variants, cytosolic release of mtDNA, its sensing by the cGAS‐STING pathway and the subsequent production of cytokines and chemokines promoting the initiation of the auto‐immune response against melanocytes; collectively providing evidence that vitiligo is a mitochondrial disease (Figure 4K). These results have a direct impact on clinical management of vitiligo patients since vitiligo melanocytes presenting with numerous mtDNA variants appear as predictive biomarkers of response to mitochondrial‐specific treatments, opening up new avenues in combat against anti‐melanocytic immunity in vitiligo patients.

## AUTHOR CONTRIBUTIONS

Ana C. B. Sant'Anna‐Silva, Thomas Botton and Meri K. Tulic performed and analysed the experiments and with Thierry Passeron wrote the manuscript. Andrea Rossi, Jochen Dobner and Nguyen Thach performed the melanocyte sequencing, Lauriane Blot and Hanene Bzioueche measured ROS production in menadione experiments, Konrad Kleszczynski and Kerstin Steinbrink provided advice on EM imaging and protocols for ROS measurements, Sophie Pagnotta performed EM experiments and Nathalie M. Mazure helped with interpretation of images. Stéphane Rocchi provided reagents, manuscript feedback and funding support for Ana C. B. Sant'Anna‐Silva. Jean Krutmann provided intellectual input and direction of sequencing experiments. Thierry Passeron and Meri K. Tulic conceived, supervised and funded the study. All authors read and gave feedback on the final version of the manuscript.

## CONFLICT OF INTEREST STATEMENT

Thierry Passeron is a consultant for AbbVie, Almirall, Amgen, Bristol Myers Squibb, Calypso, Galderma, Incyte Corporation, Janssen, Eli Lilly, Novartis, Pfizer, Roivant, UCB and VYNE Therapeutics; has received grants and/or honoraria from AbbVie, ACM Pharma, Almirall, Amgen, Astellas, Bristol Myers Squibb, Calypso, Celgene, Galderma, Genzyme/Sanofi, GlaxoSmithKline, Incyte Corporation, Janssen, LEO Pharma, Eli Lilly, Novartis, Pfizer, Roivant, Sun Pharmaceuticals, UCB and VYNE Therapeutics; is the cofounder of NIKAIA Therapeutics; and has patents on WNT agonists or GSK3b antagonist for re‐pigmentation of vitiligo and with Meri K. Tulic has a patent for the use of CXCR3B blockers in vitiligo. Meri K. Tulic is a consultant for Pfizer and ISIS Pharma. Jean Krutmann is a consultant for AbbVie and has received grants and/or honoraria from AbbVie.

## ETHICS STATEMENT

The study was approved by the Regional Ethics Committee CPP Sud‐Est VI, 1429 (N12.034) and conducted in accordance with The Code of Ethics of the World Medical Association (Declaration of Helsinki).

## Supporting information

Supporting Information

## Data Availability

All data are available, upon reasonable request, from the corresponding authors.
