# Supplementary material for: Vitiligo auto‐immune response upon oxidative stress‐related mitochondrial DNA release opens up new therapeutic strategies
Source: Clin Transl Med. 2024 Aug 7;14(8):e1810. doi: 10.1002/ctm2.1810 (PMC11306283; doi:10.1002/ctm2.1810)
Supplement: Supplementary file 1 — Supporting Information [file CTM2-14-e1810-s001.docx]

**Supplementary information**

**LETTER-TO-EDITOR WITH PREVIOUS SUBMISSION NUMBER: CTM2-2024-05-1844**

**Vitiligo auto-immune response upon oxidative stress-related mitochondrial DNA release opens up new therapeutic strategies**

Ana C. B. Sant’Anna-Silva^1*^, Thomas Botton^1*^, Andrea Rossi^2^, Jochen Dobner^2^, Hanene Bzioueche^1^, Nguyen Thach^2^, Lauriane Blot^1^, Sophie Pagnotta^3^, Konrad Kleszczynski^4^, Kerstin Steinbrink^4^, Nathalie M. Mazure^1^, Stéphane Rocchi^1^, Jean Krutmann^2,5^, Thierry Passeron^1,6^*^§^, Meri K. Tulic^1^*^§^ * These authors contributed equally, ^§^ Corresponding authors

^1^ Université Côte d’Azur, INSERM U1065, C3M, Nice, France

^2^ IUF-Leibniz Research Institute for Environmental Medicine, Düsseldorf, Germany

^3^ Common Centre of Applied Microscopy (CCMA), Université Côte d’Azur, Nice, France

^4^ Department of Dermatology, University Hospital Münster, Münster, Germany

^5^ Medical Faculty, Heinrich-Heine-University, Düsseldorf, Germany

^6^ Université Côte d’Azur, CHU Nice, Department of Dermatology. Nice, France

**Corresponding authors:**

Dr Meri K. Tulic. INSERM U1065, C3M, 151 route St Antoine de Ginestière 06200 Nice, France. Email: [meri.tulic@unice.fr](mailto:meri.tulic@unice.fr) ; Phone number: +33 762 451 284

Prof. Dr. Thierry Passeron. Department of Dermatology. Archet 2 Hospital. CHU Nice. 151 route St Antoine de Ginestière 06200 Nice, France. Email: [thierry.passeron@unice.fr](mailto:thierry.passeron@unice.fr); Phone number: +33 4 92 03 64 88

**MATERIAL AND METHODS**

***Patients***

Non-segmental vitiligo patients (n=16) were recruited from the Dermatology Department of the L’Archet Hospital, Nice CHU, and were enrolled in the study after informed, written consent was obtained. Healthy donors (n=14) surveyed for suspicion of skin cancer or familial history but negative on examination were recruited from the same clinic. The two groups were matched for age and sex, and vitiligo patients had no other auto-immune diseases **(Supplementary Fig. 1)**. Sample collection consisted of a 4-mm skin punch biopsy from the non-lesional sites of vitiligo patients which was used for the isolation of melanocytes and 10 mL blood used for the isolation of peripheral blood mononuclear cells (PBMCs) by Ficoll gradient centrifugation (Lymphoprep®, Euromedex, France). The PBMCs were frozen in FCS 10 % DMSO and kept at −156 °C until subsequent analysis. The study was approved by the Regional Ethics Committee CPP Sud‐Est VI, 1429 (N12.034) and conducted in accordance with The Code of Ethics of the World Medical Association (Declaration of Helsinki).

***Isolation of primary human melanocytes***

Upon arrival, skin biopsies were rinsed in 70 % ethanol followed by two times PBS 1 % Antibiotic-Antimycotic solution (Gibco) washes, prior to dissociation of dermal-epidermal junctions by overnight digestion in a Dispase solution (Life Technologies, Waltham, MA) at 4 °C. The next day, dermis was discarded and epidermis digested in a trypsin/EDTA solution for 20 min at 37 °C. Cellular suspension was passed through a 70 μm filter and melanocytes resuspended in MCDB 153 medium (Sigma-Aldrich, St. Louis, MO, USA) supplemented with 2 % foetal bovine serum (FBS; Hyclone Perbio, Brevieres, France), 5 μg/ml insulin (Sigma-Aldrich), 0.5 μg/ml hydrocortisone (Sigma-Aldrich), 16 nM tetradecanoylphorbol-13-acetate (TPA) (Sigma-Aldrich), 1 ng/ml fibroblast growth factor (FGF; Promega, Madison, WI, USA), 15 μg/ml bovine pituitary extract (Invitrogen, Waltham, MA, USA) and 10 μM forskolin (Sigma-Aldrich). Melanocytes were maintained at 37 °C in a humidified 5 % CO_2_ atmosphere and supplemented with 0.08 % G418 geneticin (20 μg/ml, Invitrogen) for ~2 weeks to eliminate rapidly growing cells (i.e., keratinocytes hence selecting for melanocyte propagation). Once selected, melanocytes were grown in Cascade Biologics 254 medium (ThermoFisher Scientific, Waltham, MA) with Human Melanocyte Growth Supplement 1 (HMGS, Gibco).

***Mitochondrial DNA sequencing***

mtDNA enrichment was performed through amplification of the mitochondrial genome using PCR, generating nine overlapping amplicons^1-4^. These fragments were then sequenced on an Oxford Nanopore Technologies (ONT) MinION device with a Flongle adaptor and flow cell, following a previously established protocol^5^. First, we pooled the PCR products and prepared a sequencing library using a ligation kit and barcodes (ONT). The library was then loaded onto a Flongle flow cell and sequenced for 24 h^6^. After sequencing, the fast5 files were basecalled using the guppy basecaller with specific parameters^7^. The resulting FASTQ files were concatenated, aligned to the mtDNA revised Cambridge Reference Sequence (rCRS) using minimap2, and variant calls were identified using Mutserve with a threshold of 0.05 or directly in Mitopore. Only mtDNA variants present in at least 12.5 % of reads were considered.

***Immunofluorescence Microscopy***

Cells were grown on glass coverslips, washed with PBS, fixed with a 3.7 % formaldehyde solution in PBS containing 250 nM HEPES for 15-min at room temperature, rinsed 3 times 5-min with ice-cold PBS, permeabilized for 3-min with 1% Triton X-100, rinsed another 5 times 5-min in room temperature PBS, blocked for 30-min with a 10% FBS solution in PBS and incubated with primary antibodies against dsDNA (PROGEN AC-30-10 at 1/500), TOM20 (BD Biosciences BD612278 at 1/500) or p-TBK1 Ser172 (Cell Signalling Technologies #5483 at 1/150). This was followed by appropriate secondary antibodies including Alexa Fluor 594 goat anti-mouse IgG (H+L) (A11032), Texas-Red goat anti-rabbit IgG (H+L) (T6391) and Alexa Fluor 488 goat anti-Mouse IgM (SA5-10150) (Life Technologies). Nuclei were labelled with Hoechst 33342 (Sigma-Aldrich). The coverslips were mounted on glass slides using ProLong® Diamond Antifade Mountant (Life Technologies). The samples were visualized using Nikon A1R confocal microscope on an inverted Nikon Eclipse Ti stand (Nikon Instruments, Japan) using objectives Plan Apochromat 63x/ 1.4 oil NA and Argon LASER 488 nm and/or DPSS 561 nm. The microscope was composed of 2 PMTs equipped with 450/50 and 700/75 filter set, 2 GaAsP equipped with 515/30 and 585/65 filter-set, and 1 external PMT for transmission. Z-acquisitions were performed using the microscope z-drive each 0.3 µm. Images were processed by FiJi software. Quantification of p-TBK1 foci was performed on at least 45 cells *per* melanocyte culture. A p-TBK1 focus was defined as a bright spot visible on at least two consecutive z-slices. A cell was considered positive if presenting at least three p-TBK1 foci.

***Measurement of dsDNA and mitochondrial markers by QPCR in cultured supernatants***

dsDNA was measured using Nanodrop (Thermo Fisher Scientific) and results expressed as ng/μl. MT-ND1 mRNA and MT-CO1 mRNA were measured by qPCR. Initially, RNA was extracted using RNeasy kits (Qiagen, Düsseldorf, Germany). One μg RNA was used to synthesize cDNA using the Reverse Transcription System (Promega) which was then used as a template for amplification by real-time qPCR with SYBR^TM^ Green reagent (Life Technologies, CA, USA) and specific primers directed against MT-ND1 and MT-CO1 (primer sequences are given in **Supplementary Table 1**). All measurements were performed in triplicate and results normalized to the expression of the 18S rDNA housekeeping gene.

***Electron Microscopy***

Melanocytes were seeded in 12-well culture plates. Following confluency, cells were washed twice in PBS and then fixed with 1.6 % glutaraldehyde in phosphate buffer (0.1 M pH 7.4) for 1 hr at room temperature. They were then rinsed in cacodylate buffer (0.1 M pH 7.4) and post-fixed in 1% osmium tetroxide (reduced with 1% potassium ferrocyanide). After being rinsed in distilled water, cells were gradually dehydrated in ethanol, embedded in epoxy resin, and incubated at 60°C overnight for polymerization. Ultrathin sections (80 nm) were assembled on copper grids and contrasted with lanthanides salts (gadolinium and samarium) followed by lead citrate. Sections were observed under a JEOL JEM 1400 electron microscope equipped with a Morada SIS camera. Mitochondrial mass was assessed using Mitochondria Analyzer plugin in ImageJ software (version 2.1) and degree of mitochondrial branching was scored semi-quantitatively from EM images.

***ATP Content***

The steady-state intracellular ATP content was measured using the CellTiter-Glo® Luminescent Cell Viability Assay (Promega; Madison, WI, USA) following the manufacturer’s instructions. Mitochondrial ATP and glycolytic ATP were measured upon treatment with oligomycin (ATP synthase inhibitor, 5 μM) or 2-deoxy-glucose (hexokinase inhibitor, 10mM). Luminescence was monitored with a Thermo Scientific™ Multiskan™ FC Microplate Photometer and values were calculated based on an ATP standard curve. Data were normalized to cell count.

***Oxygen consumption rate***

The oxygen consumption rate (OCR) was measured using a Seahorse XF96 extracellular flux analyser (Agilent). Cells were seeded at an initial concentration of 1 × 10^4^ cells/well of a XF96 plate, and they were allowed to adhere for 24 h. A measurement plate containing calibrant solution (100 μl per well) was placed in a CO_2_-free incubator at 37 °C overnight. The next day, this plate was run as calibration. Meanwhile, culture media were removed, cells washed twice with PBS and 100 μl of fresh media was added containing 5 mM glucose, 1 mM pyruvate and 4 mM glutamine. To eliminate CO_2_ residue in the medium, cells were incubated for 1 h *prior* to the experiment at 37 °C with CO_2_ in a non-humidified incubator. After calibration, OCR was assayed by sequential addition of mitochondrial inhibitors and uncoupler. Following stabilization of basal respiration, ATP-synthase inhibitor oligomycin (1 µM, port A) was initially added to evaluate the oxygen consumption independent of mitochondrial ATP production. Then, the protonophore carbonyl cyanide m-chlorophenyl hydrazone (CCCP) was added, forcing the transport of H^+^ throughout the mitochondrial inner-membrane and reaching the maximum mitochondrial respiration (500 nM, port B and C). Finally, 0.5 μM rotenone (inhibitor of CI) plus antimycin A (inhibitor of CIII) were added (port D) to block mitochondrial respiration. At least 6-technical replicates were done in the same plate for each sample and 6-measurements were carried out at baseline and after each injection. Three independent experiments were performed. The OCR value was normalized to cell numbers *per* well.

***Reactive oxygen species (ROS) production***

At approximately 70 % confluency, primary human melanocytes were treated with 50 µM menadione A (Sigma-Aldrich) (or not treated as controls) for 1 h at 37 °C. Cells were then collected, washed with PBS, and stained with 5 µM dihydrorhodamine for 30 min at 37 °C. Finally, ROS-positive cells were analyzed using BD FACSCalibur (BD, Pont-de-Claix, France) and analysis quantified using FlowJo software (version 10.8.2).

***DNA extraction***

Genomic DNA isolation was performed using the DNeasy Blood and Tissue Kit (Qiagen) according to the manufacturer's protocol.

***Whole exome sequencing (WES)***

WES was performed at BGI Genomics (Warszawa, Poland) using their standardized protocol. Briefly, upon arrival, DNA was quantified with the Qubit dsDNA BR Assay system (Life Technologies) and its quality was assessed by 1% agarose gel electrophoresis. After random fragmentation of genomic DNA using Covaris S-220, fragmented DNA was selected by Agencourt AMPure XP-Medium kit to an average size of 200-400 bp. The selected fragments were then run through end-repair, 3’-adenylation, adapters-ligation and PCR amplification followed by AxyPrep Mag PCR clean up. Hybrid capture was performed using SureSelect Human All Exon v.6 followed by another AxyPrep Mag PCR clean up. The double-stranded PCR products were heat-denatured and circularized by the splint oligo sequence forming the single strand circle DNA (ssCir DNA) as the final library. The library was amplified to make DNA nanoballs (DNBs) which have more than 300 copies of one molecular. The DNBs were loaded into the patterned nanoarray and paired 100 bases reads were generated in the way of sequenced by combinatorial Probe-Anchor Synthesis (cPAS) on DNBSEQ-G400 platform. After filtering with SOAPnuke, over 75 million clean reads were detected in each sample with an average Q30 of 95.26 %. Alignment on hg38 was performed with BWA v.0.7.17. On average, we obtained a 68.02x mean sequencing depth on the whole genome excluding gap regions. SNP and indel calling, and annotation were performed using GATK v.4.1.4.1. A special emphasis was placed on the analysis of genes involved in antioxidant mechanisms.

***Catalase activity***

Catalase activity was measured in lysates of 2 ×10^6^ PBMCs using the Catalase Activity Assay Kit from Abcam according to the manufacturer's protocol.

***Protein Extraction and Immunoblotting***

Approximately 2.5 × 10^5^ cells were seeded *per* 6-well plates. 24 h after incubation, cells were washed with PBS and 100 µl of 1xRIPA buffer (Sigma-Aldrich) with protease and phosphatase inhibitors (Merck) were added in each well. Cells were scraped-off, collected and frozen at −80 °C until further use. For protein extraction and immunoblotting, cell lysates were thawed, and proteins were quantified by Pierce BCA protein assay kit. LDS Sample Buffer (Thermo Fisher Scientific) was added, and sample volume was adjusted to equal protein concentration. Samples were heated at 90 ^°^C for 5 min and 15–20 µg of protein extract *per l*ane was loaded on SDS-PAGE gel. After running at 100 V for 1.5 h, the proteins were transferred onto a polyvinylidene fluoride membrane (Millipore) to a blotting membrane. The proteins were incubated with primary antibodies overnight at 4 °C using the following series of antibodies: total OXPHOS WB Antibody Cocktail (1:1,000 Abcam #ab110413) containing the antibodies anti-NDUFB8, anti-SDHB, anti-UQCRC2, anti-COX II and anti-ATP5 as a premixed solution; vinculin (1:1,000 Cell Signaling #4650); IRF7 (1:1,000 Cell Signaling #4920); IRF3 (1:1,000 Cell Signaling #4302); NF-κB p65 (1:1,000 Cell Signaling #8242); NF-κB p100/p52 (1:1,000 Cell Signaling #3017). The next day, membranes were incubated for 1h at 4 ^°^C with the appropriate HRP-conjugated secondary antibodies: anti-rabbit HRP conjugated secondary (1:3,000 Thermo Fisher Scientific) or anti-mouse HRP conjugated secondary (1:3,000 Thermo Fisher Scientific). Proteins were detected with an ECL System from Amersham.

***Immune Function***

Secretion of CXCL-9, CXCL-10, CCL-19, IL-1β, IL-18, IFNα and IFNβ were measured under basal condition or after pre-treatment of melanocytes with the Nrf2 activators DMF (50 μM, Selleckchem) or NK-252 (100 μM, Targetmol); the non-specific antioxidant vitamin C (50 μM, Sigma) or cytosolic antioxidant recombinant SOD1 (50 μg/ml, Abcam); the mitochondrial-specific antioxidant recombinant SuperOxide Dismutase SOD2 (50 μg/ml, Abcam); the VDAC-1 oligomerization inhibitor VBIT-4 (20 μM, Selleckchem); or the TBK1 inhibitor GSK8612 (10 μM, MedChem Express) using commercially available ELISA kits (PeproTech, USA and R&D Systems Quantikine ELISA, Lille France). After a 2-h pre-treatment with the indicated compound or recombinant protein, medium was changed to regular culture medium. Culture supernatants were collected for secretomes analysis 24 h later.

***Migration Experiments***

The ability of the secretomes of recombinant SOD2-, or GSK8612-pretreated melanocytes from healthy individuals, Vitiligo LV or HV patients to attract PBMCs from either healthy or vitiligo subjects was tested using a Cytoselect 96-well Cell Migration Assay (Cell Biolabs, San Diego, CA, USA). Migratory cells were quantified using CyQuant GR Fluorescent Dye and results were compared to secretomes from non-pretreated melanocytes from the same patients.

***Statistical analyses***

Statistical analyses were performed with GraphPad Prism® 6.0 software (La Jolla, CA, USA). Normality of data and homogeneity of variances was assessed using Shapiro-Wilk and Bartlett tests, respectively. Statistical significance was determined by unpaired Student’s t-test comparing two independent groups. Multiple groups comparison was performed using ordinary one-way or two-way ANOVA with appropriate *post-hoc* analysis (Mann-Whitney U to test unpaired differences between groups and Wilcoxon signed rank test for paired differences). Statistical significance was considered at α  ≥  0.05. All experiments were performed with a minimum of 3 biological replicates, and whenever possible, 2 or more technical replicates. All data represent mean ± SD.

**REFERENCES**

1. Ramos, A. M. et al. Design of a high-density SNP genotyping assay in the pig using SNPs identified and characterized by next generation sequencing technology. PLoS One **4** (2009). e6524.

2. Zascavage, R. R., Thorson, K. & Planz, J. V. Nanopore sequencing: An enrichment-free alternative to mitochondrial DNA sequencing. *Electrophoresis* **40**, 272-280 (2019).

3. Dobner, J. et al. Mitochondrial DNA integrity and metabolome profile are preserved in the human induced pluripotent stem cell reference line KOLF2.1J. *Stem Cell Rep*. **19**, 343-350 (2024).

4. Dobner, J. et al. mtDNA Analysis using Mitopore. *MTMCD* **101231** (2024).

5. Nguyen, T., Ramachandran, H., Martins, S., Krutmann, J. & Rossi, A. Identification of genome edited cells using CRISPRnano. *Nucleic Acids Res.* **50**, W199-W203 (2022).

6. Li, H. Minimap2: Pairwise alignment for nucleotide sequences. *Bioinformatics* **34**, 3094-3100 (2018).

7. Wickham, H. *ggplot2* *Elegant Graphics for Data Analysis.* (Springer Nature, Houston, 2016).

**
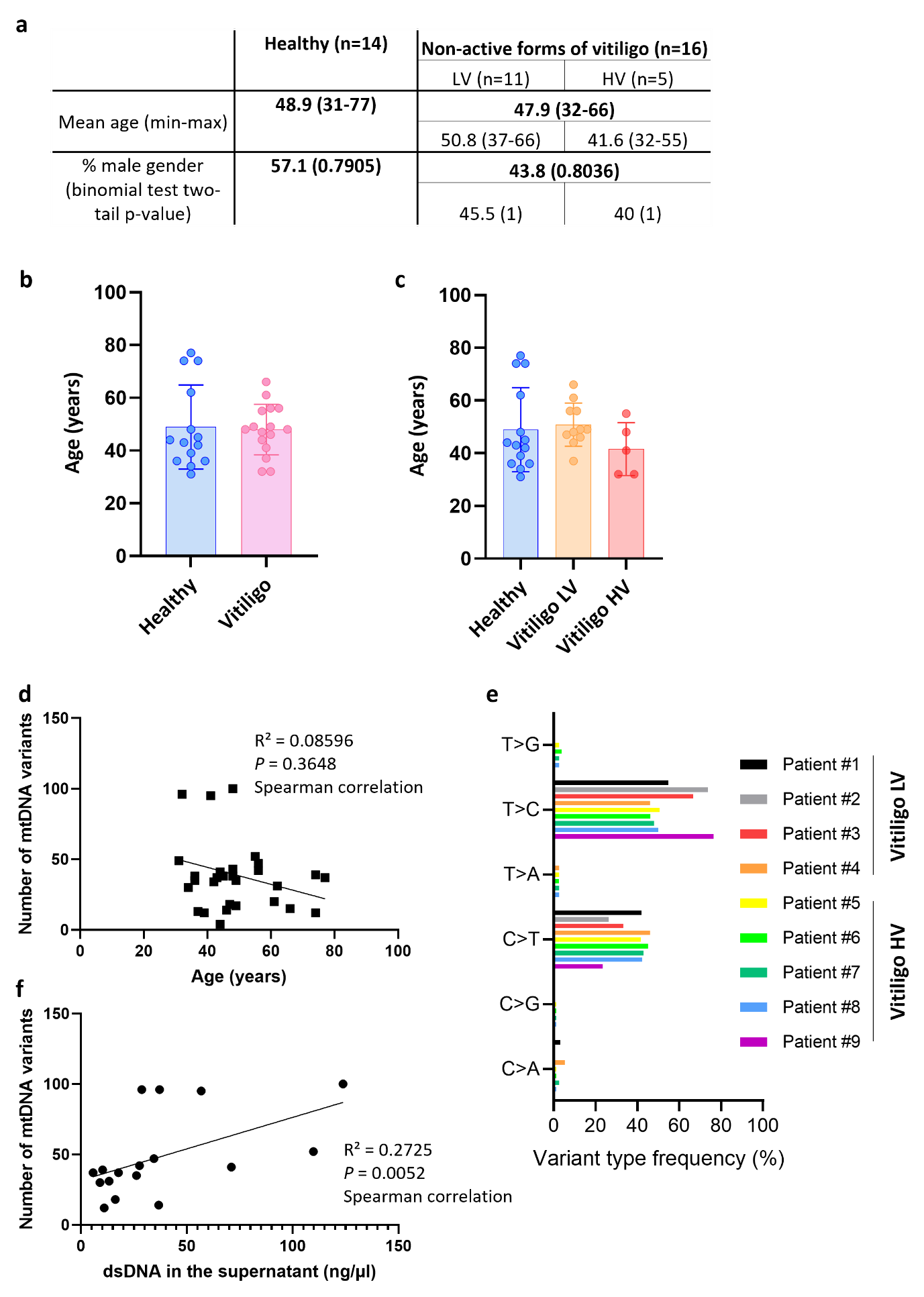
**

**Supplementary Fig. 1. Cohort description**

**(a)** Table summarizing the characteristics of the healthy donors and non-active vitiligo subjects from whom the primary melanocytes cultures used in the study have been established; **(bc)** Comparison of the age of the subjects between healthy and vitiligo subjects (B) or between healthy, Vitiligo LV and Vitiligo HV (C); **(d)** Correlation between the age of the subjects and the number of mtDNA variants measured in their primary melanocyte cultures. **(e)** Variant type frequency measured in mtDNA. **(f)** Correlation between the number of mtDNA variants and dsDNA measured in melanocyte culture supernatant. All data represent mean ± SD. One-way ANOVA compared to healthy controls.

**
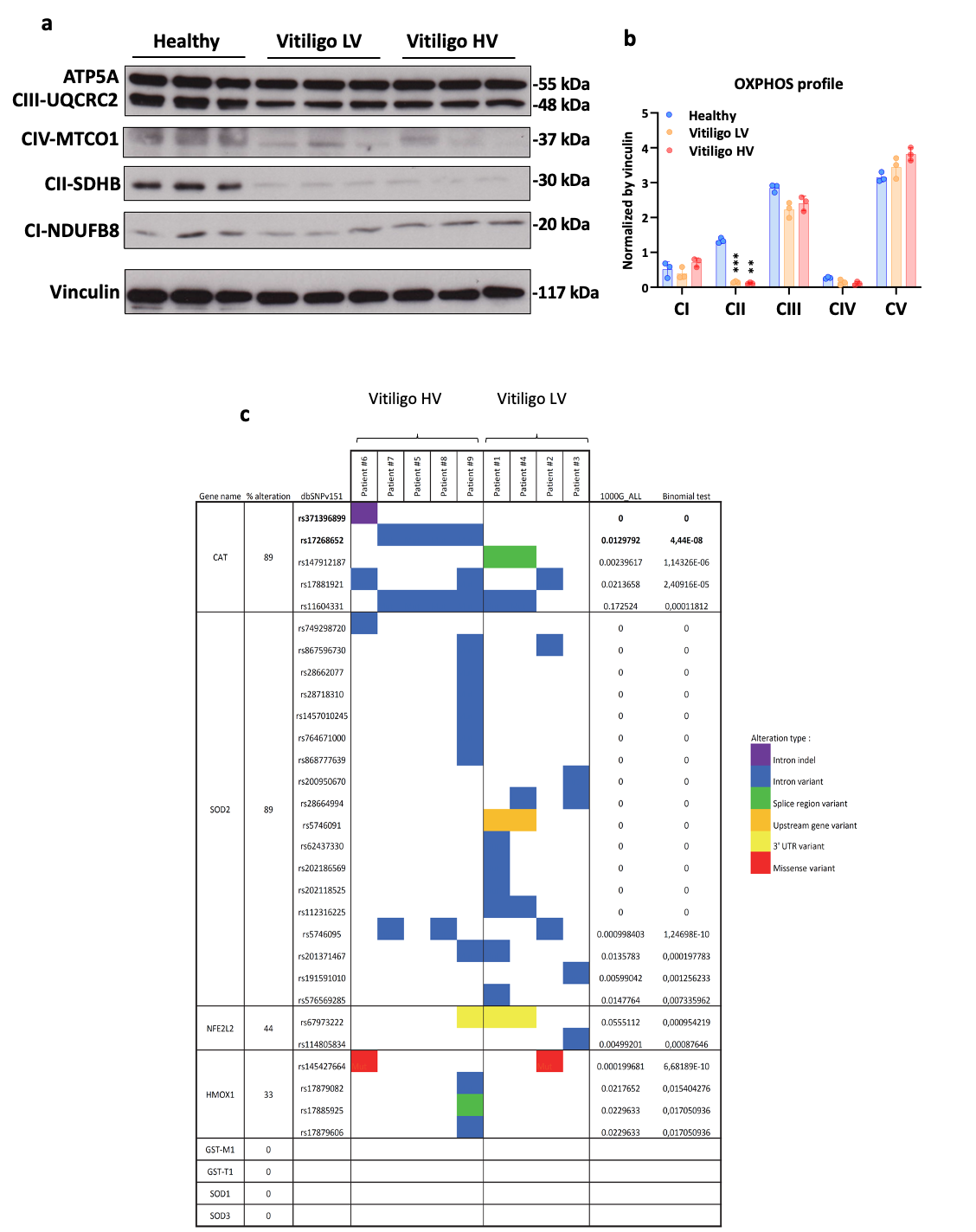
**

**Supplementary Fig. 2. Vitiligo samples exhibit lower mitochondrial complexes subunits expression and are associated with uncommon SNPs in catalase.**

**(a)** Immunoblot analysis of subunits of electron transfer system complexes NDUFB8 (CI), SDHB (CII), UQCRC2 (CIII), MTCO1 (CIV), ATP5A (ATP synthase) and **(b)** its quantification. Vinculin was used as a loading control (n = 3). All data represent mean ± SD. Two-way ANOVA for multiple comparisons ***P* < 0.01, *** *P* < 0.001. **(c)** Oxidative stress genes polymorphism was monitored by whole exome sequencing (WES).

**
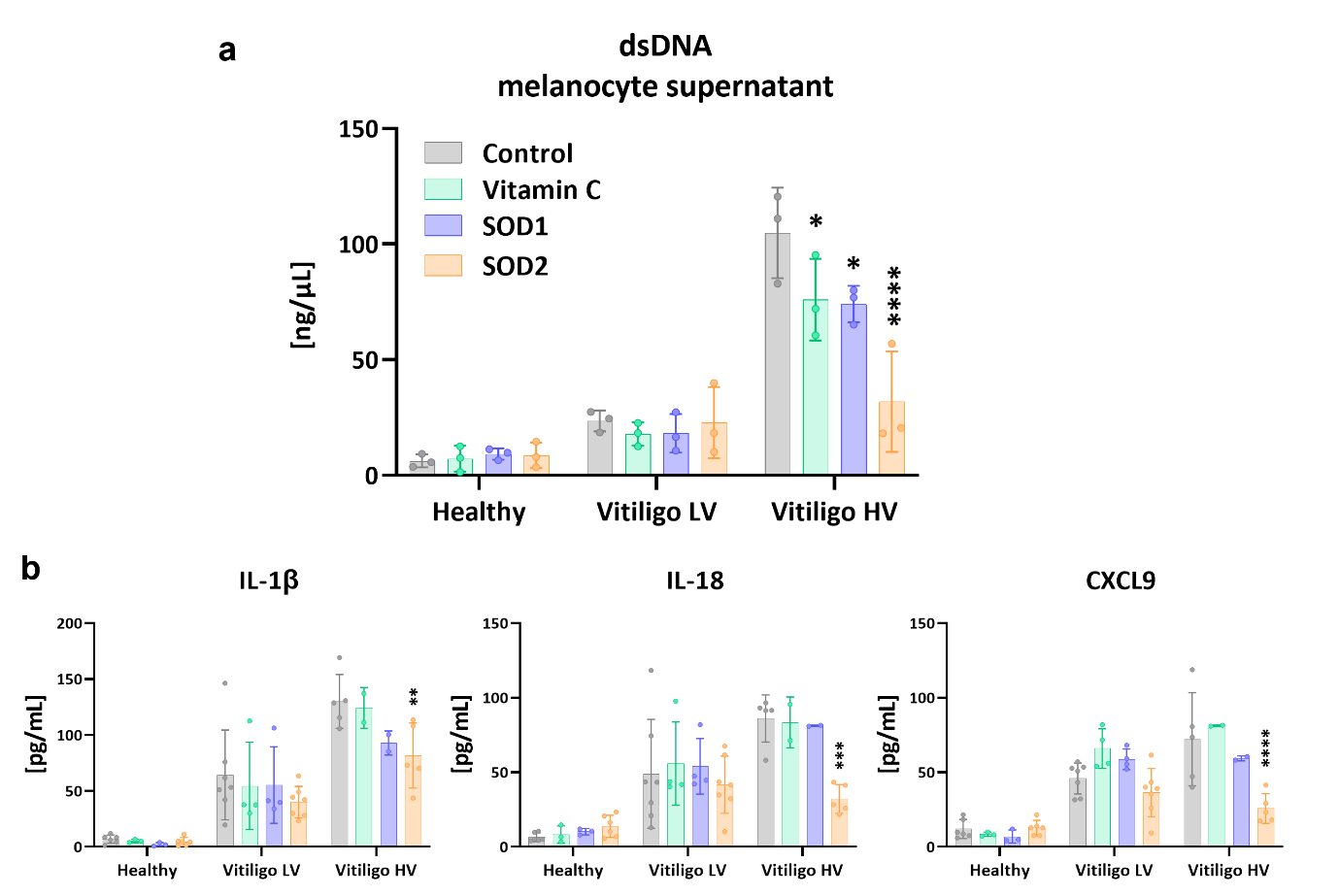
**

**Supplementary Fig. 3. Mitochondrial ROS scavenging undermines inflammatory response in Vitiligo HV melanocytes.**

**(a)** Measurements of dsDNA and **(b)** IL-1β, IL-18 and CXCL10 in the supernatant of Vitiligo HV melanocytes treated with vitamin C, recombinant SOD1 or SOD2. All data represent mean ± SD. Two-way ANOVA compared to healthy controls. **P* < 0.05; ***P* < 0.01; ****P* < 0.001; *****P* < 0.0001.

**Supplementary Table 1. Primer sequences**

| **Gene​** | **Primer sequence​** |
| --- | --- |
| *MT-ND1*​ | Forward: 5′-CCACCTCTAGCCTAGCCGTTTA-3’​ |
|  | Reverse: 5′-GGGTCATGATGGCAGGAGTAAT-3’​ |
| *MT-CO1*​ | Forward: 5′-GACGTAGACACACGAGCATATTTCA-3’​ |
|  | Reverse: 5′-AGGACATAGTGGAAGTGAGCTACAAC-3’​ |
| *18S rDNA*​ | Forward: 5’-TAGAGGGACAAGTGGCGTTC-3’​ |
|  | Reverse: 5’-CGCTGAGCCAGTCAGTGT-3’​ |
